# [^18^F]Fluciclovine PET discrimination between high- and low-grade gliomas

**DOI:** 10.1186/s13550-018-0415-3

**Published:** 2018-07-25

**Authors:** Ephraim E. Parent, Marc Benayoun, Ijeoma Ibeanu, Jeffrey J. Olson, Constantinos G. Hadjipanayis, Daniel J. Brat, Vikram Adhikarla, Jonathon Nye, David M. Schuster, Mark M. Goodman

**Affiliations:** 1 Department of Radiology and Imaging Sciences, Emory University School of Medicine, 1841 Clifton Rd. NE, 2nd floor, Atlanta, GA 30329 USA; 2 Department of Radiology, Massachusetts General Hospital, Boston, MA USA; 3 Department of Radiology, Texas Tech University Health Sciences Center Foster School of Medicine, El Paso, TX USA; 4 Department of Neurosurgery, Emory University School of Medicine, Atlanta, GA USA; 5 Department of Neurosurgery, Mount Sinai Beth Israel, New York, NY USA; 6 Department of Pathology, Northwestern University Feinberg School of Medicine, Chicago, IL USA; 7 Department of Information Sciences, City of Hope National Medical Center, Duarte, CA USA

**Keywords:** ^18^F-fluciclovine, Glioma, Amino acid, PET

## Abstract

**Background:**

The ability to accurately and non-invasively distinguish high-grade glioma from low-grade glioma remains a challenge despite advances in molecular and magnetic resonance imaging. We investigated the ability of fluciclovine (^18^F) PET as a means to identify and distinguish these lesions in patients with known gliomas and to correlate uptake with Ki-67.

**Results:**

Sixteen patients with a total of 18 newly diagnosed low-grade gliomas (*n* = 6) and high grade gliomas (*n* = 12) underwent fluciclovine PET imaging after histopathologic assessment. Fluciclovine PET analysis comprised tumor SUV_max_ and SUV_mean_, as well as metabolic tumor thresholds (1.3*, 1.6*, 1.9*) to normal brain background (TB_max_, and TB_mean_). Comparison was additionally made to the proliferative status of the tumor as indicated by Ki-67 values.

Fluciclovine uptake greater than normal brain parenchyma was found in all lesions studied. Time activity curves demonstrated statistically apparent flattening of the curves for both high-grade gliomas and low-grade gliomas starting 30 min after injection, suggesting an influx/efflux equilibrium. The best semiquantitative metric in discriminating HGG from LGG was obtained utilizing a metabolic 1 tumor threshold of 1.3* contralateral normal brain parenchyma uptake to create a tumor: background (TB_mean1.3_) cutoff of 2.15 with an overall sensitivity of 97.5% and specificity of 95.5%. Additionally, using a SUV_max_ > 4.3 cutoff gave a sensitivity of 90.9% and specificity of 97.5%. Tumor SUV_mean_ and tumor SUV_max_ as a ratio to mean normal contralateral brain were both found to be less relevant predictors of tumor grade. Both SUV_max_ (*R* = 0.71, *p* = 0.0227) and TB_mean_ (TB_mean1.3_: *R* = 0.81, *p* = 0.00081) had a high correlation with the tumor proliferative index Ki-67.

**Conclusions:**

Fluciclovine PET produces high-contrast images between both low-grade and high grade gliomas and normal brain by visual and semiquantitative analysis. Fluciclovine PET appears to discriminate between low-grade glioma and high-grade glioma, but must be validated with a larger sample size.

**Electronic supplementary material:**

The online version of this article (10.1186/s13550-018-0415-3) contains supplementary material, which is available to authorized users.

## Background

Primary brain and central nervous system tumors are relatively uncommon with 23,800 estimated new cases in the USA in 2017 resulting in 16,700 deaths [1]. High-grade gliomas (HGG; WHO grades III and IV) in particular carry a dismal prognosis [2]. Conventional magnetic resonance imaging (MRI) represents the standard of care for biopsy, surgical resection, and radiation planning for brain tumors. Yet, dependence on MRI alone can lead to inaccurate grading of the tumor and insufficient resection of highly aggressive lesions. Approximately 40% of HGGs demonstrate no enhancement, while low-grade gliomas (LGG; WHO grade II) infection and post treatment effects may demonstrate nonspecific enhancement [3]. Furthermore, tumors initially felt to be LGGs, due to lack of MRI contrast enhancement, may in fact be HGGs due to the presence of malignant, anaplastic foci. Thus, molecular imaging with positron emission tomography (PET) has allure providing specific information that can improve tumor grading and delineation.

The non-natural amino acid PET radiotracer, *anti*-1-amino-3-^18^F-fluorocyclobutane-1-carboxylic acid (FACBC, fluciclovine (^18^F)) was approved by the Food and Drug Administration (FDA) in May 2016 for the indication of suspected recurrent prostate cancer in patients with elevated PSA after therapy, and the radiotracer was also granted orphan drug status for brain gliomas [4]. As first described in 1999 by Shoup et al., fluciclovine has been shown to have increased uptake in brain gliomas in subsequent pre-clinical and clinical studies [5–10]. In distinction to other amino acid PET tracers, such as L-[methyl-^11^C]-methionine (MET), ^18^F fluoro-ethyltyrosine fluoroethyltyrosine (FET), and 3,4-dihydroxy-6-[^18^F] fluoro-L-phenylalanine (FDOPA) which are predominantly transported via system L amino acid transporters, fluciclovine undergoes cellular transport by both system L and system alanine-serine-cysteine (ASC), specifically ASCT2, in human astrocytes and glioma cells [11]. Both system L and ASCT2 are known to be obligate amino acid exchanges with intracellular and extracellular substrates exchanged in a 1:1 manner [12]. However, ASCT2 is not expressed at the luminal side of the blood-brain-barrier (BBB) and is therefore likely not involved with the transport of fluciclovine into the normal brain and in tumors with an intact BBB [13].

The goal of this study was to evaluate the ability of fluciclovine PET to discriminate between HGGs and LGGs. We hypothesized that quantitative metrics such as SUV_max_, tumor to background ratio, and time activity curve (TAC) analysis would reflect relative differences of amino acid transport in both classes of tumor and therefore have suitable diagnostic performance for differentiating HGGs and LGGs. We also hypothesized fluciclovine uptake would correlate with the degree of nuclear proliferation as expressed via MIB-1 (Ki-67 labelling index).

## Methods

### Subject recruitment

Patients with biopsy proven glial neoplasms were recruited from February 19, 2008, to April 21, 2015, under a National Institutes of Health grant (R01CA121320). The recruitment protocol was approved by the Institutional Review Board (IRB) and complied with the Health Insurance Portability and Accountability Act (HIPPA). The radiotracer was administered under FDA Investigational New Drug (IND) 72,437 and was synthesized either via automated synthesis [14] or the FastLab Cassette System (GE Healthcare). Safety monitoring during the drug infusion was performed, and no adverse events were recorded. In total, 51 patients were recruited with 37 eventually receiving at least one fluciclovine PET study post-histologic confirmation as required by the IRB. For this analysis, patients were sub-selected under the inclusion criteria of biopsy proven disease either after stereotactic brain biopsy or excisional biopsy/partial resection. Patients with systemic chemotherapy or radiation before the fluciclovine PET in the current episode of care were excluded, resulting in a total of 16 patients included in this study (Table 1). We excluded that group of patients from this analysis as we felt that the previously treated high-grade glioma patients were too heterogenous in their prior therapy which may introduce too many confounding errors into the image analysis. Additionally, no patients were imaged with recurrent previously treated low-grade glioma (LGG). Two of the included 16 patients had two discrete lesions each that underwent tissue sampling and PET lesion analysis resulting in a total of 18 lesions. In 1 patient, a bis-chloroethylnitrosourea (BCNU) wafer had been employed during excisional biopsy/partial resection as per existing standard of care.

**Table 1:** Patient demographics

Lesion	Patient sex	Age	Histopathology	WHO grade	Histologic verification (days before PET)	Ki-67 (%)
1	F	58	Oligodendroglioma	II	SB (4 day)	3
2	M	38	Oligodendroglioma	II	SB (19 day)	3
3	M	56	Diffuse astrocytoma	II	SB (13 day)	4
4	M	36	Oligodendroglioma	II	SB (14 day)	< 1
5	M	39	Oligodendroglioma	II	EB (42 day)	N/A
6	F	32	Diffuse astrocytoma	II	EB (61 day)	4
7	M	45	Anaplastic astrocytoma	III	EB (31 day)	40
8	F	50	Glioblastoma	IV	SB (7 day)	12
9	M	70	Glioblastoma	IV	SB (15 day)	40
10^a^	F	72	Glioblastoma	IV	SB (27 day)	30
11^a^	F	72	Glioblastoma	IV	SB (27 day)	30
12	M	58	Glioblastoma	IV	EB (3 day)	N/A
13^a^	F	36	Glioblastoma	IV	EB (13 day)	N/A
14^a^	F	36	Glioblastoma	IV	EB (13 day)	N/A
15	F	58	Glioblastoma	IV	EB (14 day)	10
16	F	65	Glioblastoma	IV	EB (16 day)	35
17	M	67	Glioblastoma	IV	EB (13 day)	8
18	F	60	Glioblastoma	IV	EB (18 day)	N/A

*SB* stereotactic biopsy, *EB* excisional biopsy/partial resection, *N/A* not available

^a^Indicates lesions from the same patient

### Image acquisition

PET imaging of the head was performed using a high resolution research tomograph (HRRT) scanner (Siemens Medical Solutions, Knoxville, TN, USA). Patients were administered 366–399 MBq (9.9–10.8 mCi) fluciclovine (^18^F) intravenously via pump over 4 min. Continuous dynamic PET imaging of a single bed position was begun at the beginning of injection and ended at the 65 min time point. Attenuation scanning was performed in single mode with a 30-mCi ^137^Cs point source. The data was collected in list mode and later histogrammed into 15 time points (6 × 30, 4 × 180, and 5 × 600 s). PET emission data were corrected for attenuation, random, and scatter and reconstructed with an ordinary Poisson ordered–subset expectation maximization (OP-OSEM) algorithm (6 iteration, 16 subsets) to a 256 × 256 × 207 image matrix (voxel size 1.2 mm^3^) [15]. Image data was post-reconstruction smoothed with a 24 mm FWHM Gaussian filter to reduce noise and improve contrast in the brainstem. The final isotropic resolution was 4.6 mm and matched that reported in our previous work. Data was transferred to a MIM workstation (MIM Software, OH) for further analysis.

### Selection of regions of interest (ROIs)

A board-certified radiologist using the Absolute Threshold Contouring Tool (MIM Software, OH, USA) drew regions of interest (ROIs) over the tumors and background ROIs (i.e., contralateral brain and venous confluence) for all time points.

**Fig. 1:**
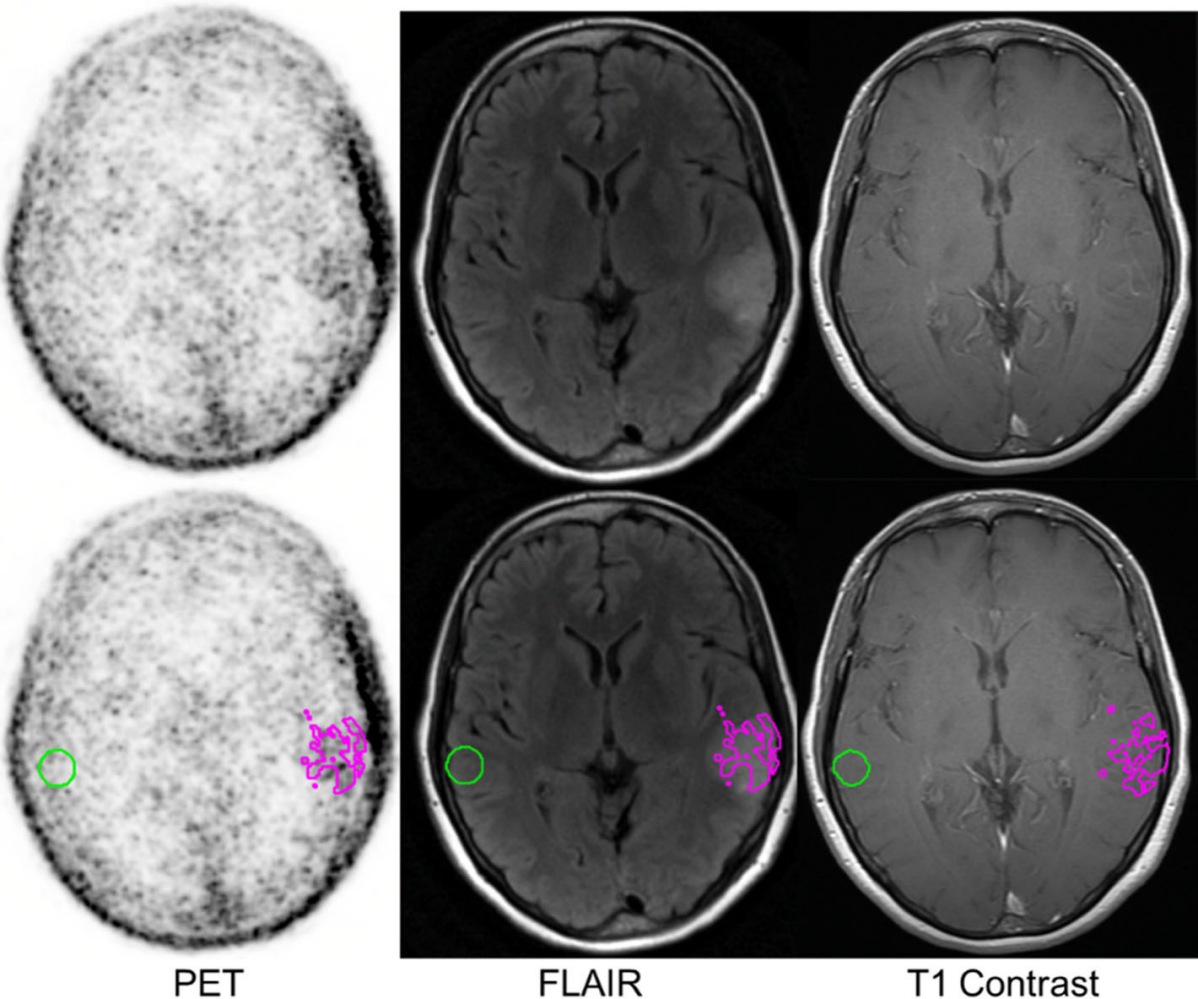
Fluciclovine PET at 30 min post-injection of oligodendroglioma with regions overlaid on MRI. Green sphere is the area of normal brain fluciclovine uptake. Magenta area is the metabolic tumor uptake defined as 1.3* contralateral normal brain uptake (TB_mean1.3_)

**Fig. 2:**
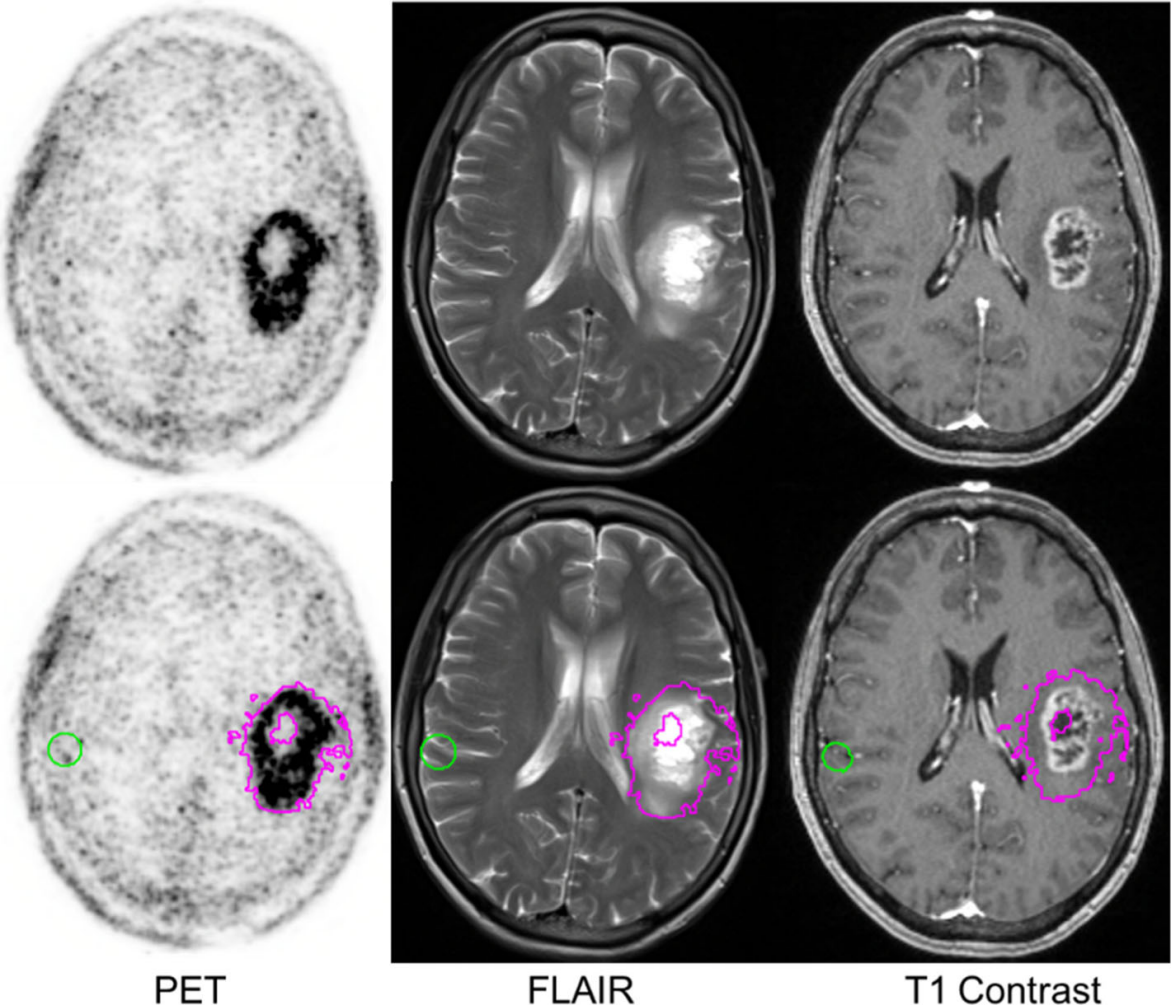
Fluciclovine PET at 30 min post-injection of glioblastoma fused with regions overlaid on MRI. Green sphere is the area of normal brain fluciclovine uptake. Magenta area is the metabolic tumor uptake defined as 1.3* contralateral normal brain uptake (TB_mean1.3_)

Fluciclovine PET images were co-registered to T1 post contrast MRI and FLAIR sequences. Tumor regions of interest (ROIs) were defined by creating a spherical PET ROI to include the volume of tissue demonstrating hyperintense FLAIR signal corresponding to the biopsy proven glioma. Within this PET ROI, the voxels with peak activity were used to derive a tumor maximum standardized uptake value (SUV_max_). A 15-mm spherical ROI was placed over the contralateral normal brain, including both gray and white matter, to provide a normal mean standardized uptake value (SUV_mean_). The SUV_mean_ of the contralateral normal brain was utilized to select a threshold for defining metabolically active tumor within the aforementioned PET ROI. Minimum thresholds of 1.3*, 1.6*, and 1.9* of the contralateral normal parenchymal SUV_mean_ were used to define the tumor SUV_mean_ (Figs. 1 and 2). These thresholds were selected according to similar work done with FET and other amino acid PET tracers [16, 17]. Careful consideration when drawing ROIs over the tumor was used to exclude blood pool or adjacent choroid plexus which could falsely contribute to metabolic tumor volume.

### Time activity curves (TACs)

Time activity curves for each lesion, normal contralateral parenchyma, and venous confluence were obtained by averaging PET metrics such as SUV_max_ or tumor: background (TB_mean_) over all lesions at each time point using Excel (Microsoft, WA, USA). Differences between HGGs, LGGs, blood pool, and normal brain parenchymal SUVs, along with assessment of apparent TAC equilibrium kinetics, were detected using visual inspection with statistical comparison.

### Semiquantitative PET metrics

SUV_max_ and SUV_mean_ for each tumor lesion, contralateral normal background, and venous confluence were recorded at all time points. Tumor to background ratios for each lesion were calculated as TB_max_ = (SUV_max_ tumor)/(SUV_mean_ background) and TB_mean_ = (SUV_mean_ tumor)/(SUV_mean_ background) at all imaged time points and at all thresholds relative to normal contralateral brain resulting in TB_mean1.3_, TB_mean1.6_, and TB_mean1.9_.

### Estimating threshold values for classification of HGG versus LGG for most relevant predictor variables

The optimal threshold for differentiating HGGs from LGGs utilizing tumor SUV_max_ was calculated using a receiver operator characteristic curve (ROC) for each tumor SUV_max_ measurements from 30 to 50 min post-injection, when there was an apparent plateau of the SUV_max_. A single patient with anaplastic astrocytoma was included within the high-grade group for the purposes of this analysis. Sensitivity and specificity for identifying HGGs is reported based on the optimal threshold. A similar approach using ROC curves was applied to each TB_mean_ dataset at least 30 min post-injection to obtain a TB_mean_ threshold to distinguish HGG from LGG.

### Ki-67 staining and imaging correlation

Five micron sections of formalin-fixed, deparaffinized tissue from 13 of the 18 lesions were immunostained using antibodies against Ki-67 (clone MIB1, 1:160 dilution, DAKO Corp, Carpinteria, CA, USA) using normal brain as the control. Five lesions did not undergo proliferative analysis. Negative controls were run simultaneously and had primary antibody replaced with buffer. Antigen retrieval was conducted in citrate buffer at pH 6.0 under a pressure of 15 lbs/in^^2^ for 3 min. Envision+ Dual Link Kit (DAKO Corp) was used as the detection system, with diaminobenzidine as the chromogen and hematoxylin as the counterstain. Staining was performed with the DAKO Autostainer.

### Statistical analysis

Statistical analysis was performed with MATLAB R2017a prerelease (Mathworks, MA) using the Statistics and Machine Learning Toolbox. Four semiquantitative [^18^F]-fluciclovine PET metrics were assessed as potential discriminators of HGG vs LGG: SUV_max_, SUV_mean_, TB_max_, and TB_mean_. Each metric was analyzed at every time point demonstrating apparent equilibrium kinetics (i.e., 30, 40, 50, and 60 min post-injection). Relative importance of each predictor was made using a lasso regularization of a logistic regression model to identify relevant predictor variables (see Additional file 1). Unbalanced 2-way ANOVA followed by post hoc Tukey’s test were also used to compare lesion SUV_max_, normal brain parenchyma SUV_mean_, and venous confluence SUV_mean_ at equilibrium time points.

**Fig. 3:**
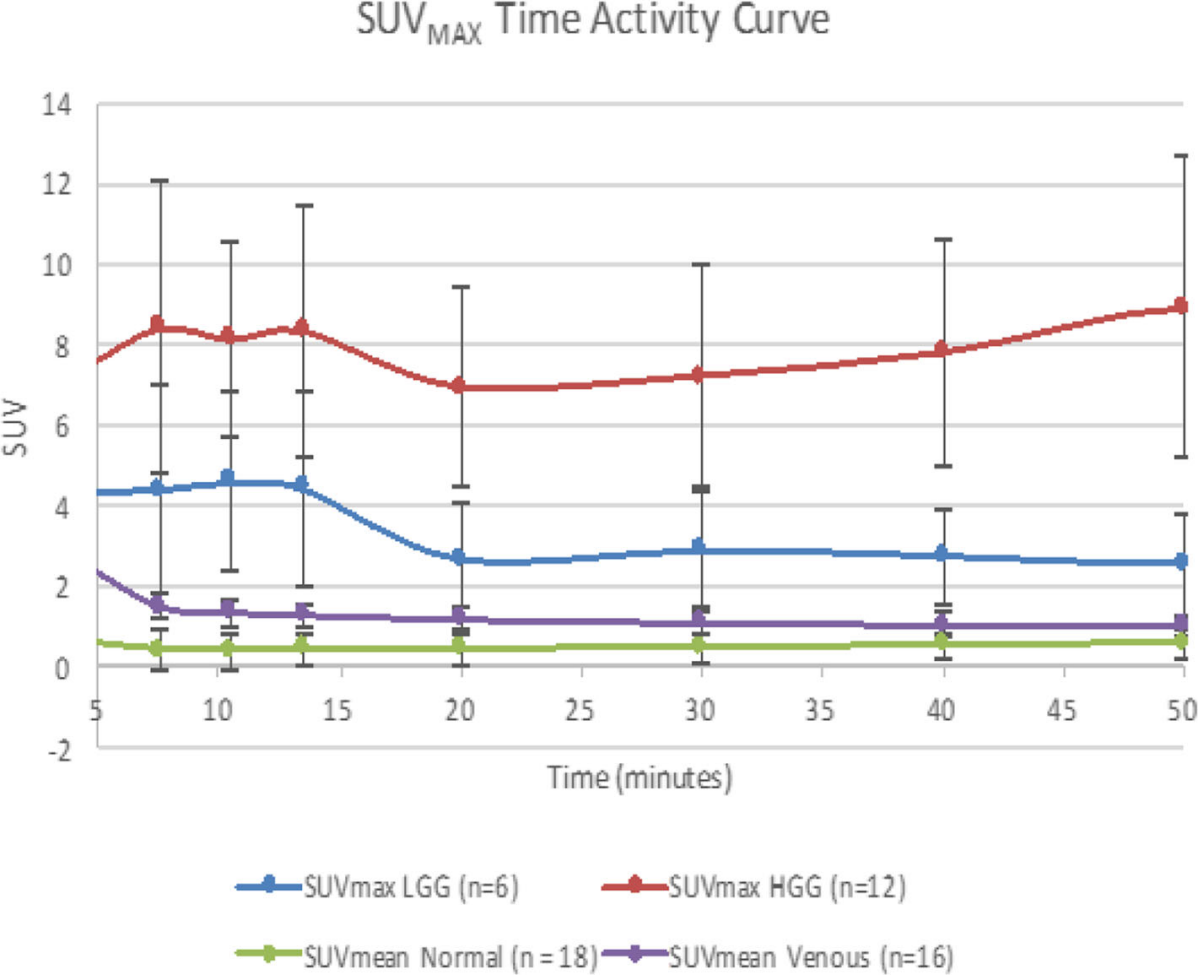
Time activity curves of HGG (red) and LGG SUV_max_ (blue) show equilibrium kinetics starting 30 min post-injection. Statistically significant differences are noted between HGG and LGG, normal brain parenchyma SUV_mean_ (green), and venous blood pool SUV_mean_ (purple). Each lesion was compared to the contralateral normal

**Fig. 4:**
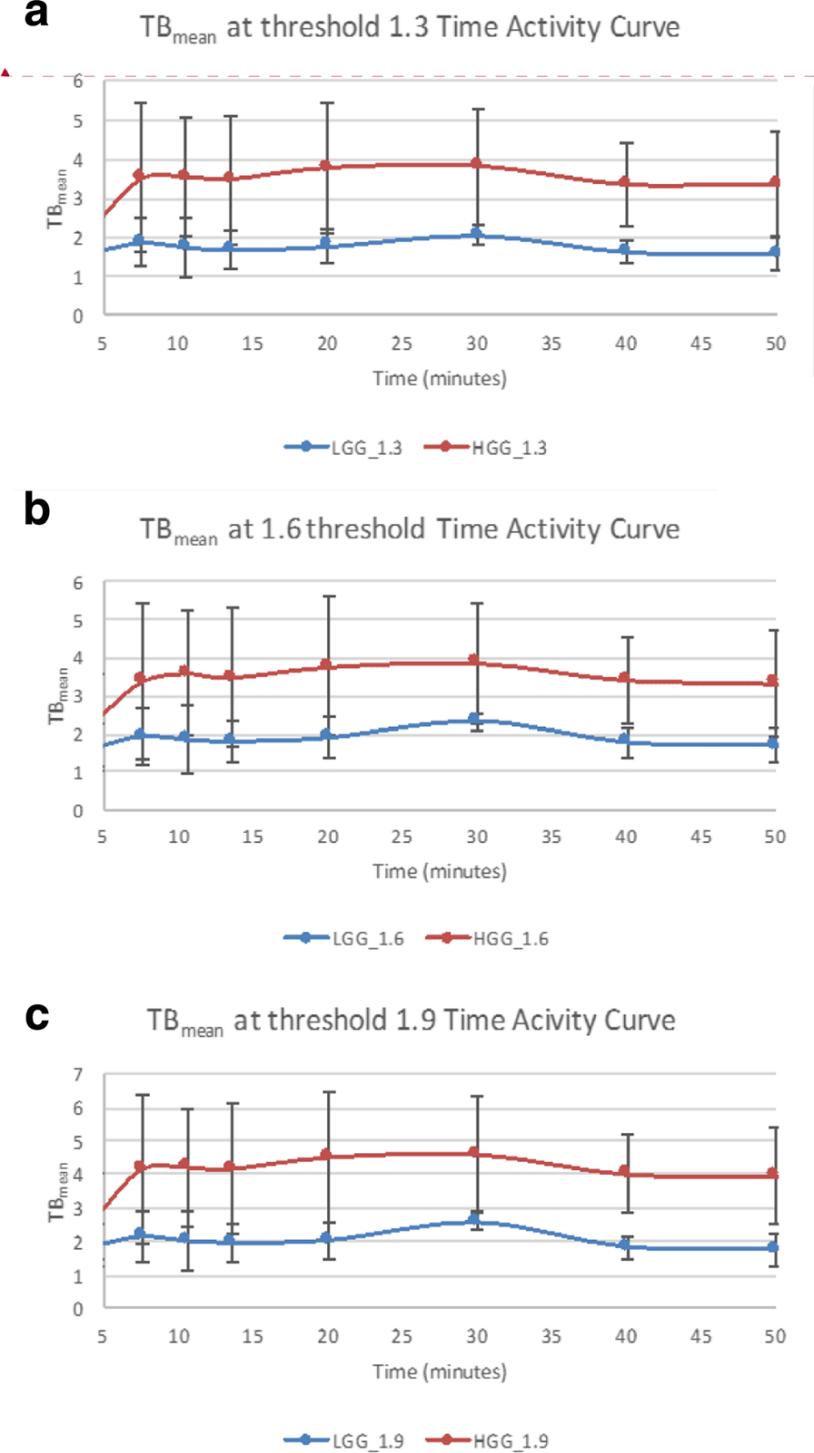
Time activity curves using TBmean for HGG (red) and LGG (blue) with **a** threshold of 1.3 × SUV_mean_ normal brain parenchyma, **b** 1.6 × SUV_mean_ normal brain parenchyma, **c** 1.9 × SUV_mean_ normal brain parenchyma

Unbalanced statistical design was used since only 6/10 HGG patients and 5/6 LGG patients completed the full 65-min acquisition protocol. The remaining studies were prematurely halted due to patient request. This patient attrition skewed the graphs at 60 min, so although the full data set was analyzed, only data up to 50 min is displayed in Figs. 3 and 4.

Statistical analysis on single time point data using scatter plots of SUV_max_ and TB_mean_ was performed using unequal variance two-tailed *t* tests of World Health Organization (WHO) II and WHO IV tumor uptake at equilibrium time points. Only one lesion was consistent with WHO III classification and was not included in this statistical analysis. However, this lesion was included in the high-grade tumor class for purposes of ROC analysis and with the high-grade group for time activity curve analysis as previously discussed.

For expression of percent positive Ki-67 cells in the malignant lesions correlated with lesion SUV_max_ and TB_mean_ at 30 min post-injection, the linear correlation coefficient (*R* value) for strength of correlation and *p* value to test the null hypothesis of a zero slope was performed.

## Results

### Subject demographics

Sixteen patients were included per inclusion criteria (Table 1). All 16 patients (8 M and 8 F with mean age of 49.6 years) completed the fluciclovine PET scan after either having undergone a stereotactic biopsy (8 patients) or excisional biopsy/partial resection (8 patients) prior to the fluciclovine PET scan. PET imaging was performed an average of 19.4 days (3–61 days) after surgical intervention. The confirmed histopathology for each patient were distributed as follows: 4 oligodendrogliomas (WHO grade II), 2 diffuse astrocytomas (WHO grade II), 1 anaplastic astrocytoma (WHO grade III), and 11 glioblastomas (WHO grade IV) with two patients having multifocal disease involving 2 distinct lesions. One of the multifocal glioblastoma patients previously had a known oligodendroglioma which had been treated with external radiation 2 years prior to the fluciclovine PET study. In total, 18 tumors were analyzed from 16 patients.

#### Time activity curves

Time activity curves (TACs) of averaged tumor SUV_max_, contralateral parenchymal uptake, and blood pool SUV_mean_ levels are shown beginning at 4.5 min (after completion of the 4 min infusion) to focus on equilibrium kinetics (Figs. 3 and 4). The reason for the slow 4 min infusion was based on the initial desire to broaden the input function so that we could accurately measure fluciclovine levels temporally when setting up multiple frame reconstruction. This technique would make the kinetic modeling easier. Future studies are being planned with bolus injection of fluciclovine to better evaluate the early kinetic uptake profiles of high-grade gliomas.

Dynamic analysis of HGGs demonstrates flattening of the TACs suggestive of a fluciclovine influx/efflux equilibrium starting at approximately 30 min post-injection; however, this needs to be validated due to our small sample size. Though visual analysis suggests there is a rise in HGG SUV_max_ at 50 min, results of an unbalanced two-way ANOVA analysis of HGGs between 30 and 60 min found no statistically significant difference between pairwise comparisons of HGG SUV_max_ values except between 30 and 50 min and is not validated on additional semiquantitative metrics (*p* = 0.0213). Similar unbalanced two-way ANOVA analysis of LGG tumors found no statistical change of LGG SUV_max_ between 30 and 60 min (*p* = 0.3177) which may be consistent with fluciclovine influx/efflux equilibrium starting at approximately 30 min (Fig. 3).

### Semiquantitative PET metrics and threshold values for classification of HGG versus LGG for most relevant predictor variable

Logistic regression found that SUV_max_ and TB_mean_ were good discriminators of HGG versus LGG, whereas TB_max_ and SUV_mean_ were relatively irrelevant predictors of tumor grade (see Additional file 1). Using ROC analysis on all HGG and LGG SUV_max_ levels obtained at least 30 min after injection demonstrated an optimal discrimination threshold of SUV_max_ > 4.32. This SUV_max_ threshold provides a sensitivity of 90.9% and specificity of 97.5% (AUC 0.985; Table 2).

**Table 2:** Receiver operator characteristic (ROC) curve analysis

Uptake parameter	Threshold	Sensitivity (%)	Specificity (%)	Area under the curve (AUC)
SUV_max_	4.32	97.5	90.9	0.985
TB_mean1.3_	2.15	97.5	95.5	0.989
TB_mean1.6_	2.56	95	100	0.989
TB_mean1.9_	2.58	95	100	0.991

TB_mean_ of HGGs and LGGs was evaluated utilizing minimum threshold levels of 1.3*, 1.6*, and 1.9* contralateral normal brain parenchyma SUV_mean_, resulting in TB_mean1.3_, TB_mean1.6_, and TB_mean1.9_, respectively (Fig. 4). Each TB_mean_ metabolic threshold level resulted in a unique discriminator of HGG and LGG as expected. For example, setting a TB_mean1.3_ threshold to 2.15 resulted in a sensitivity of 97.5% and specificity of 95.5% (AUC 0.989) with a statistically significant difference in uptake between WHO II and WHO IV lesions (*p* = 0.0036 at 30 min, *p* = 0.00041 at 40 min, and *p* = 0.0088 at 50 min). Similar results for TB_mean1.6_ show an optimal threshold of 2.56 with sensitivity of 95.0% and specificity of 100% (AUC 0.989). Finally, ROC analysis shows an optimal threshold for TB_mean1.9_ of 2.58 with a sensitivity of 95.0% and specificity of 100% (AUC = 0.991). This analysis found that all metrics based on SUV_max_ and TB_mean_ have high discriminatory power, but SUV_max_ and TB_mean1.3_ had the highest sensitivity (lowest false negative) for detection of HGG, with TB_mean1.3_ showing higher specificity. Due to our small sample size, these metrics will need to be externally validated.

**Fig. 5:**
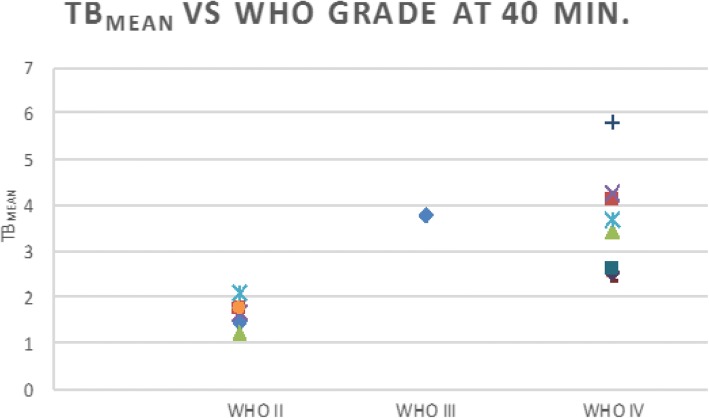
Scatter plot of individual lesion TB_mean_1.3_ of WHO II, III, and IV tumors at 40 min post-injection

Despite the small sample size, statistically significant differences between HGG and LGG SUV_max_ as well as between either HGG or LGG SUV_max_ and normal or venous SUV_mean_ were noted (for example between HGG and LGG, *p* < 0.00001) (Fig. 5). No statistically significant difference between venous blood pool and normal parenchymal SUV_mean_ was noted (*p* = 0.29).

### Ki-67 tumor proliferation

**Fig. 6:**
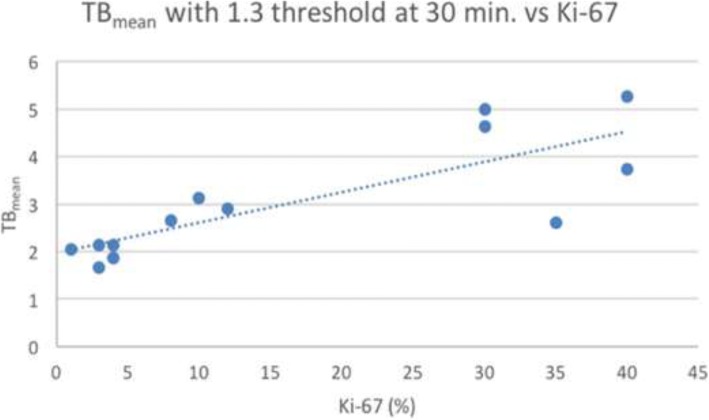
Lesion by lesion correlation between fluciclovine TB_mean_1.3_ and Ki-67

The correlation of SUV_max_ and TB_mean_ with Ki-67 from the evaluable 13 out of 18 lesions was examined. While SUV_max_ and TB_mean1.3_, TB_mean1.6_, and TB_mean1.9_ were all found to have statistical correlation with Ki-67 levels (*p* < 0.05), the strongest correlation was with TB_mean1.3_ (Fig. 6). TB_mean1.3_ has a robust correlation with Ki-67 (*R* = 0.81, *p* = 0.00081) with a slightly less robust correlation between SUV_max_ and Ki-67 values (*R* = 0.62, *p* = 0.0227). While there was a range of Ki-67 values observed in the HGG group, a very narrow range of Ki-67 values was observed in the LGG group. Of the sampled LGG, 4/5 lesions had a Ki-67 of 3 or 4 and no isolated analysis of LGG or HGG was thus performed.

## Discussion

The goal of this study was to determine if PET imaging with the synthetic amino acid radiotracer anti-1-amino-3-^18^F-fluorocyclobutane-1-carboxylic acid (FACBC, fluciclovine (^18^F)) could discriminate high-grade glioma (HGG) from low-grade glioma (LGG) tumors by defining the optimal imaging metrics. We also wanted to study if fluciclovine uptake correlates with degree of nuclear proliferation as evidenced by Ki-67 values. There is a strong relationship between the expression of Ki-67 and LAT1 in gliomas [18]. Considerable overlap of Ki-67 levels is known to exist between the different malignancy groups, and Ki-67 is an important independent prognostic factor in human astrocytomas [19]. We therefore wanted to include Ki-67 levels to help characterize the patterns of fluciclovine uptake we observed. While there are a few publications that have reported Ki-67 levels and fluciclovine PET uptake in HGGs, there are no reports to date evaluating fluciclovine uptake in LGGs.

Mutations in IDH1 and IDH2 have been known to be a key development of many gliomas with information regarding outcomes and response to therapy [20]. Of the 18 lesions studied, there were only two (oligodendroglioma, diffuse astrocytoma) that had IDH-1 mutations. The remaining lesions were either IDH-1 negative or the status was not evaluated on histopathological evaluation. We therefore did not attempt to determine the effects of IDH-1 mutation on fluciclovoine uptake.

Fluciclovine was found to be an effective PET agent in discriminating between LGGs and HGGs in regions of suspected disease by MRI imaging. All patients had accumulation of fluciclovine in areas corresponding to known tumor as evident by visual analysis as well as semiquantitative image analysis. We evaluated 4 different semiquantitative PET metrics as means to identify and discriminate fluciclovine uptake between HGGs and LGGs: SUV_max_, SUV_mean_, TB_max_, and TB_mean_. We demonstrated that SUV_max_ and TB_mean_ were effective metrics for this discrimination, while tumor SUV_mean_ and TB_max_ were not statistically robust discriminators of HGG from LGG. In addition, fluciclovine activity was correlated with Ki67.

Our findings are important since differentiating HGGs from LGGs on MRI remains a challenge, despite continued advancement in MR imaging, including MR perfusion and MR spectroscopy [21]. Metabolic imaging of brain tumors using PET has shown promise in improving tumor extent, grading, and prognosis. Normal brain parenchyma has relatively low levels of amino acid transporters, and malignant tumors typically have increased need for amino acids resulting in upregulation of amino acid transporters and metabolism. Amino acid PET tracers, such as L-[methyl-^11^C]-methionine (MET), ^18^F-fluoroethyl-L-tyrosine (FET), and 3,4-dihydroxy-6-[^18^F] fluoro-L-phenylalanine (FDOPA), all demonstrate high uptake in tumor tissue and low uptake in normal brain.

MET, FET, and FDOPA are all transported via LAT1 amino acid transporters [22]. LAT1 is widely expressed in primary human cancers and cancer cells lines [23] and is active at the blood-brain barrier [24]. MET, FET, and FDOPA have all demonstrated a positive correlation between glioma uptake and LAT1 expression [25, 26]. FET has been shown to reliably differentiate between LGG and HGG using dynamic PET, but not by using conventional static PET. FET demonstrates increasing time activity curves in LGGs, while HGGs show decreasing time activity curves after the first 5–15 min [27, 28]. Dynamic imaging is time-consuming in both image acquisition and image analysis, and most FET imaging involves summation images from 20 to 40 min [29]. These summation images are known to demonstrate a high overlap between WHO grades, limiting the accuracy for tumor grading [27, 30]. Kinetic studies involving MET and FDOPA have shown an ability to distinguish LGG from HGG in newly diagnosed gliomas, but both tracers fail to distinguish between LGG and HGG in recurrent previously treated tumors [31]. Of note, none of these PET radiopharmaceuticals have been FDA approved.

While fluciclovine is FDA approved for detection of recurrent prostate cancer, it initially was developed to detect HGGs [8]. In vivo pre-clinical PET imaging of rat gliomas reported that fluciclovine uptake correlated to tumor extent more accurately than MRI [5] and demonstrated high correlation with tumor cell density, irrespective of blood-brain barrier integrity [6]. As compared to ^18^F-FET, ^18^F-FDOPA, and ^11^C-MET, fluciclovine undergoes transport via both system L and system ASCT2. However, since ASCT2 is not expressed at the luminal side of the BBB, the relevance of this transporter in glioma imaging is uncertain, though hypothetically may be an additional pathway of uptake with disruption of the BBB that may occur with higher grade disease or intervention.

A recent report by Kondo et al. [7] evaluated the safety and efficacy of fluciclovine to detect suspected tumor in areas without contrast enhancement in patients with glioblastoma. They reported that fluciclovine uptake in glioblastomas corresponded to Ki-67 levels. Additionally, a multicenter phase IIb trail of fluciclovine brain PET involving patients with suspected HGG and LGG was performed. Semiquantitative metrics were not reported; however, the positive predictive value of visualized fluciclovine uptake in areas of suspected glioma without contrast enhancement was 100.0%. Areas demonstrating both contrast enhancement and fluciclovine uptake had a positive predictive value of 87.5% [9]. A separate study imaged six patients with suspected HGG or LGG with both fluciclovine PET and MET PET within a 2-week period [10]. The authors found that the SUV_mean_ and SUV_max_ of each lesion were higher with MET, but TB_max_ and TB_mean_ were higher with fluciclovine due to low normal brain parenchyma uptake. Only a single anaplastic astrocytoma and a single glioblastoma were included in this study, and the authors did not attempt to distinguish HGG from LGG based on their imaging characteristics.

In our study, we found that tumor SUV_max_ and TB_mean_ have a strong statistical correlation with Ki-67 in 13 of the 18 lesions imaged. Additionally, despite our small sample size, we found that the semiquantitative fluciclovine mean metabolic tumor uptake compared to contralateral normal brain is able to distinguish between LGGs and HGGs. To identify mean metabolic tumor uptake, we set a minimum metabolic threshold as a ratio to mean contralateral brain, similar to work done with other pure LAT1 PET amino acids. We empirically chose a 1.6* mean contralateral brain threshold using published work with ^18^F-FET and also examined threshold values at ± 20% [16]. Though all TB_mean_ thresholds were discriminatory, using a TB_mean1.3_ cutoff of 2.15 provided the best means to distinguish HGG from LGG among all the metrics selected when prioritizing sensitivity followed by specificity with overall sensitivity of 97.5% and specificity of 95.5% (AUC 0.989; Fig. 4). Additional analysis of fluciclovine TACs found that individual time point SUV_max_ values from 30 to 50 min were able to discriminate between HGG and LGG, using an SUV_max_ > 4.3 with a sensitivity of 90.9% and specificity of 97.5%. Note that SUV_max_ and TBR_mean,_ defined as SUV_mean_ to tumor/SUV_mean_ background, are completely independent metrics. It should be noted that SUV_max_ is highly susceptible to noise and which may explain in part the apparent different TACs between SUV_max_ and TBR_mean_ (Figs. 3 and 4). Additional work is required to determine if there are truly stable kinetics after 30 min or if the TACs of HGG and LGG diverge as implied by SUV_max_ measurements.

The current study has several limitations. The study population was small, and these results will need to be validated in a larger prospective study. Only a single patient with an anaplastic astrocytoma was evaluated, severely limiting our ability to characterize fluciclovine PET uptake in this important class of glioma and how it relates to either glioblastomas or LGGs. Additionally, the low fluciclovine uptake seen in both diffuse astrocytomas and oligodendrogliomas may be an artifact of the small sample size, thus explaining the difference between our study and previous reports of relatively high uptake of MET and FET in tumors with oligodendroglial components [28, 32]. Also, in contradistinction to other amino acid radiopharmaceuticals, such as FET and FDOPA, we found no statistically significant difference between venous blood pool and normal brain parenchymal SUV_mean_. These other amino acids are known to have higher brain parenchyma uptake compared to blood pool, and this difference may be due to our small sample size or possibly less penetrance of the intact BBB [33]. Future studies are being planned to include patients with suspected glioma as evidenced by MRI to undergo fluciclovine PET prior to surgical intervention, to better evaluate the transport of fluciclovoine PET across an intact BBB.

A second limitation is that, due to IRB requirements that all study subjects were required to have histopathological confirmation before imaging, fluciclovine PET was performed after stereotactic biopsy or surgical sampling/partial resection which may have confounded fluciclovine uptake. It has previously been shown that fluciclovine demonstrates little accumulation in granulocytes/macrophages and comparable uptake to FDG in T and B cells [34]. Thus, T/B cell uptake may result in false-positive fluciclovine uptake in this sample set, and future studies will need to be performed prior to surgical intervention to determine to what degree the observed fluciclovine uptake may in fact be due to inflammation. Ideally, in future studies, imaging will be performed before tissue sampling.

Similarly, we were unable to perform stereotactic correlation of activity to presence of histologically verified tumor or to Ki-67. In all lesions imaged, including those that underwent partial excision, there was evidence of residual tumor as determined by standard of care MRI examination. However, it is known that gliomas have a large intra-tumor heterogeneity and it is possible that the tissue samples did not reflect the highest tumor grade or mitotic index. Given the constraints by the IRB, we feel that this data provides sufficient initial evidence of a correlation between fluciclovine PET uptake and tumor malignancy. Future studies are being planned to have patients with suspected glioma as evidence by MRI undergo fluciclovine PET prior to surgical intervention with the goal to correlate stereotactic tissue samples to fluciclovine PET uptake and regional Ki-67. With the intraoperative guidance techniques increasingly available, this correlation should be more practical in the future.

Finally, this analysis was not designed to differentiate progression from pseudo-progression, determine tumor presence outside of areas of MR enhancement, or correlate findings on fluciclovine PET with overall of progression free survival. These important clinical questions, in addition to other PET metrics such as SUV_peak_, metabolic tumor volume, and kinetic analysis starting at time of bolus injection, may be studied in future trials.

## Conclusions

Fluciclovine PET produces high-contrast images potentially useful for biopsy guidance. Visual and semiquantitative analysis of fluciclovine PET appear to discriminate between low-grade glioma (LGG) and high-grade glioma (HGG) and may ultimately influence treatment decisions, though results require validation in a larger series with inclusion of additional grade 3 lesions. The best semiquantitative metric in discriminating HGG from LGG was obtained utilizing a metabolic tumor threshold of 1.3* contralateral normal brain parenchyma uptake to create a TB_mean1.3_ cutoff of 2.15. Additionally, using an SUV_max_ > 4.3 cutoff also provided high accuracy and ability to discriminate between LGG and HGG. Finally, though requiring additional study, fluciclovine PET seems to demonstrate statistical flattening of time activity curves suggestive of equilibrium kinetics after approximately 30–40 min which implies that practical static imaging may be obtained at that time after radiotracer injection. Whether the apparent fluciclovione uptake equilibrium in the gliomas over the 30–50 min range is due to a true equilibrium or rather a statistical artifact from our small sample size will need to be corroborated with additional and larger studies.

## Additional file

Additional file 1: Categorizing relevant predictors for differentiating HGGs and LGGs reducing model complexity by regularized regression. (DOCX 62 kb)
