# Supplementary material for: [18F]Fluciclovine PET discrimination between high- and low-grade gliomas
Source: EJNMMI Res. 2018 Jul 25;8:67. doi: 10.1186/s13550-018-0415-3 (PMC6060188; doi:10.1186/s13550-018-0415-3)
Supplement: Supplementary file 1 — Categorizing relevant predictors for differentiating HGGs and LGGs reducing model complexity by regularized regression. (DOCX 62 kb) [file 13550_2018_415_MOESM1_ESM.docx]

**[^18^F]Fluciclovine PET discrimination between high- and low-grade gliomas.**

(For: European Journal of Nuclear Medicine and Molecular Imaging)

Ephraim E. Parent^1^*, Marc Benayoun^2^*, Ijeoma Ibeanu^3^, Jeffrey Olson^4^, Constantinos G Hadjipanayis^5^, Daniel J. Brat^6^, Vikram Adhikarla^7^, Jonathon Nye^1^, David M. Schuster^1^, Mark M. Goodman^1^.

* these authors contributed equally to the work.

^1^Department of Radiology and Imaging Sciences, Emory University School of Medicine, Atlanta, Georgia.

^2^Department of Radiology, Massachusetts General Hospital, Boston, Massachusetts.

^3^Department of Radiology, Texas Tech University Health Sciences Center Foster School of Medicine, El Paso, Texas.

^4^Department of Neurosurgery, Emory University School of Medicine, Atlanta, Georgia.

^5^ Department of Neurosurgery, Mount Sinai Beth Israel, New York, NY

^6^Department of Pathology, Northwestern University Feinberg School of Medicine, Chicago, IL.

^7^Department of Information Sciences, City of Hope National Medical Center, Duarte, CA

Address Correspondence to:

Mark M. Goodman

Department of Radiology and Imaging Sciences

Emory University School of Medicine

1841 Clifton Rd. NE, 2nd floor

Atlanta, GA 30329

[mgoodma@emory.edu](mailto:mgoodma@emory.edu)

404-727-9366

**Additional file 1 Categorizing relevant predictors for differentiating HGGs and LGGs Reducing model complexity by regularized regression**

In total, 4 semiquantitative PET metrics (SUV_max_, SUV_mean_, TB_max_, TB_mean_) were obtained for 18 lesions at multiple time points between 30-60 minutes post injection. TB_mean_ at 1.6x contralateral normal brain parenchyma was initially used. If data from all time points would have been available, this would have resulted in 72 data points (18 lesions * 4 time points) for regression analysis. Only 62 data points were available, as some patients did not complete the PET acquisition. Given the relatively small size of the data and large number of potentially relevant predictors, there was concern for potentially overfitting the data with a traditional logistic regression approach, so an L_1_-regularized logistic regression (LASSO) with 10-point cross validation was first employed, as has been successful in previous medical image feature classifiers[1]. This approach allows one to examine the trade-off between excluding predictors from the logistic regression analysis to decrease model complexity and the potential improved fit of the model to the data by including additional predictor variables.

Results indicated that minimizing classification error using the regularized regression analysis could be achieved while completely ignoring SUV_mean_ (see Figure A1). Note that the regression coefficient for SUV_mean_ (red line) is 0 when the model best matches the data (vertically-oriented, dotted green line), indicating no loss in goodness of fit when excluding this predictor. Also, note that the relatively larger coefficients for SUV_max_ and TB_mean_ over a wide range of model complexities suggest that these may be the most relevant predictors for classifying HGGs from LGGs.

**Relevant predictor variables and semi-standardized regression coefficients**

Having determined that SUV_mean_ can be excluded from the logistic regression without loss of model fit, we investigated the relative importance of the remaining predictor variables to classifying the lesions as HGG vs LGG. Although metrics for relative importance of predictor variables in logistic regression is an active area of research with no clear best method, a commonly used approach to calculate semi-standardized logistic regression coefficients has been proposed. In this method, the change in assigned probability of HGG vs LGG for a one standard deviation change in the predictor variable is calculated to determine which predictor variables more strongly influence the classification of HGG vs LGG. Results from this standardization reveal the strongest contributions from SUV_max_ and TB_mean_ with less relevance for TB_max_.

Figure A1. Trace plot of regression coefficients fit by LASSO logistic regression at different levels of model complexity.

Figure A1: B1 corresponds to SUV_max_, B2 to SUV_mean_, B3 to TB_max_, and B4 to TB_mean_.

**Using SUV_max_ and TB_mean_ to classify HGGs vs LGGs**

Having determined that SUV_max_ and TB_mean_1.3_ (see Results section of main paper) were independently the most relevant predictors in classifying HGGs and LGGs based on accuracy and sensitivity, a classic logistic regression model was tested with these two predictors to evaluate the ability of the combined information to classify the lesions as HGG vs LGG. First, the data set was separated into a training data set and a test data set by selecting at random 2 LGGs and 2 HGGs to be reserved for the test data set (multifocal HGGs were excluded from selection to prevent the training set from containing information correlated with the test set). This ultimately resulted in 14/62 data points reserved for testing and the remaining 48/62 for training the logistic regression classifier, which allowed us to examine generalizability of the classifier to new data. Results of this traditional logistic regression analysis showed 100% sensitivity and specificity on the training data with threshold determined by ROC (AUC = 1), which when generalized to include the test data revealed a sensitivity of 100% and specificity of 95.5%, using the following rule:

45.0*SUV_max_+76.0*TB_mean_1.3_ > 371.5 implies HGG (otherwise LGG).

1. Gimenez F, Xu J, Liu Y, Liu T, Beaulieu C, Rubin D, et al. Automatic annotation of radiological observations in liver CT images. AMIA Annu Symp Proc. 2012;2012:257-63.
